# How Contaminated Is the Surgical Field in Reverse Total Shoulder Arthroplasty? A Preliminary Quantitative Intraoperative Microbiological Study

**DOI:** 10.3390/jcm15135160

**Published:** 2026-07-02

**Authors:** Enrico Bellato, Michela Bersia, Francesca Menotti, Fabio Longo, Davide Blonna, Gabriele Vasario, Silvia Cortese, Eleonora Maniscalco, Lucrezia Massobrio, Paola Dalmasso, Giuliana Banche, Cristina Costa, Valeria Allizond, Filippo Castoldi

**Affiliations:** 1 Department of Surgical Sciences, University of Torino, 10126 Turin, Italy; enrico.bellato@unito.it (E.B.); silvia.cortese@unito.it (S.C.); filippo.castoldi@unito.it (F.C.); 2 Department of Public Health and Pediatrics, University of Torino, 10126 Turin, Italy; michela.bersia@unito.it (M.B.); francesca.menotti@unito.it (F.M.); f.longo@unito.it (F.L.); eleonora.maniscalco@unito.it (E.M.); lucrezia.massobrio@unito.it (L.M.); paola.dalmasso@unito.it (P.D.); cristina.costa@unito.it (C.C.); 3 Orthopaedics and Traumatology Unit, Mauriziano Umberto I Hospital, 10128 Turin, Italy; davide.blonna@gmail.com; 4 Orthopaedics and Traumatology Unit, Presidio CTO, Città della Salute e della Scienza, 10126 Turin, Italy; gabriele.vasario@gmail.com

**Keywords:** reverse total shoulder arthroplasty, surgical field contamination, *Cutibacterium acnes*, coagulase-negative staphylococci, bacterial load, periprosthetic joint infection, time to positivity

## Abstract

**Background/Objectives**: Bacterial contamination of the surgical field during shoulder arthroplasty may contribute to periprosthetic joint infection (PJI), yet data on intraoperative bacterial load and its clinical correlates remain limited. This study aimed to evaluate culture positivity and bacterial load in specimens collected at the end of reverse total shoulder arthroplasty (RTSA) and to explore their association with patient-related factors. **Methods**: Fifty-five patients undergoing elective RTSA were consecutively enrolled. At the end of surgery, three specimens per patient (two prosthetic swabs and one periprosthetic tissue sample) were collected for qualitative and quantitative microbiological analysis. Associations between bacterial load and clinical variables were assessed using mixed-effects linear regression models, while time to culture positivity was analysed using mixed-effects Cox regression models. **Results**: Among 165 specimens, *Cutibacterium acnes* was isolated in 42.4% and coagulase-negative staphylococci in 29.1%. *C. acnes* showed significantly higher bacterial loads (1.38 × 10^3^ CFU/mL) compared with aerobic bacteria (6.54 × 10^1^ CFU/mL). Higher *C. acnes* load was associated with male sex, older age, higher body mass index, smoking, and cuff tear arthropathy, whereas massive rotator cuff tear and longer time to positivity were inversely associated. Aerobic bacterial load was primarily associated with longer surgical duration. Time to positivity was shorter for aerobes than for *C. acnes*. **Conclusions**: Intraoperative bacterial contamination during RTSA is frequent and characterized by marked differences in bacterial load and growth kinetics. Quantitative assessment of bacterial burden may improve the interpretation of unexpected positive cultures.

## 1. Introduction

During shoulder arthroplasty, resident skin bacteria may contaminate deeper tissues intraoperatively. While such contamination does not inevitably result in infection, progression to prosthetic joint infection (PJI) may be facilitated by factors such as antimicrobial resistance and biofilm formation, which enhance bacterial persistence and tolerance to host defenses and antimicrobial therapy [1,2,3,4,5,6,7,8,9]. The microorganisms most commonly implicated in shoulder PJIs following elective arthroplasty are Gram-positive bacteria, primarily *Cutibacterium acnes*—an anaerobic, lipophilic bacillus inhabiting sebaceous glands—and coagulase-negative staphylococci (CoNS), with *Staphylococcus aureus* reported less frequently [4,8,10,11,12,13,14,15,16]. Although *C. acnes* and CoNS are generally considered low-virulence pathogens, distinguishing intraoperative contamination, colonization, and true infection remains particularly challenging in shoulder surgery [5,15,17,18].

These microorganisms are frequently isolated from intraoperative specimens, particularly during revision procedures. However, the clinical significance of positive intraoperative cultures remains debated, as bacterial detection does not necessarily imply active infection. Several studies suggest that both intraoperative contamination and the subsequent risk of PJI may be influenced by patient-related factors—such as sex, age, body mass index, and comorbidities—as well as by procedure- and implant-related variables [3,6,7,8,9,15,19].

Matsen and colleagues demonstrated that *C. acnes* and CoNS are the predominant bacteria detected on the shoulder skin before surgery and after skin disinfection. In contrast, other common skin commensals are rarely recovered from the incised dermis [6]. Consistently, other studies have reported *C. acnes* positivity rates of approximately 40% in deep intraoperative samples and about 20% in cultures collected at the end of shoulder arthroplasty [5,7,9,20,21].

Studies based on superficial skin sampling have shown that *C. acnes* is commonly present in the shoulder region, with a bacterial load of approximately 10^3^ CFU considered clinically relevant [12]. Moreover, *C. acnes* positivity has been shown to correlate with bacterial load and time to culture positivity [22]. However, quantitative data on the overall bacterial burden and positivity rates in the intraoperative surgical field remain lacking.

While several studies have reported culture positivity rates in shoulder surgery, quantitative data on intraoperative bacterial burden in primary reverse total shoulder arthroplasty (RTSA) remain limited, despite the increasing use of this procedure and the ongoing controversy regarding the interpretation of unexpected positive intraoperative cultures. Although intraoperative contamination does not inevitably lead to PJI, the detection of unexpected positive cultures may influence postoperative management decisions, including antibiotic therapy and follow-up strategies. For this reason, quantitative assessment of bacterial load may provide additional information beyond simple culture positivity and help refine the understanding of intraoperative contamination in this specific surgical setting. The present study, therefore, aimed to assess culture positivity and bacterial load in specimens collected at the end of RTSA and to investigate their association with patient characteristics potentially relevant to intraoperative microbial contamination.

## 2. Patients and Methods

### 2.1. Patients and Study Design

From April 2023 to April 2024, 55 patients (age range 52–85 years) undergoing primary RTSA were enrolled at San Luigi Gonzaga Hospital (Orbassano, Turin), Mauriziano-Umberto I Hospital (Turin), and Città della Salute e della Scienza Hospital (Turin). All patients provided informed consent. Patients affected by acute proximal humeral fractures, osteonecrosis, tumor, previous failed surgical treatments (either open or arthroscopic), and patients who underwent antibiotic therapy within two weeks prior to surgery, or infiltrative therapy referred to both intra-articular and subacromial corticosteroid injections within six months prior to surgery were excluded from the present study. For all the patients, demographics (i.e., age, sex, weight, height, BMI, comorbidities, indication for surgery, subacromial/intra-articular injection before RTSA) and RTSA surgery characteristics (i.e., intra- and/or postoperative complications, duration of surgery) were collected.

### 2.2. Surgical Treatment

The night before surgery, patients showered with chlorhexidine, and hair removal from the surgical area was performed on the day of surgery. Cefazolin was used as standard antibiotic prophylaxis and replaced by vancomycin in case of allergy. In the operating room, skin preparation followed a double disinfection protocol as previously described [23]; before draping, the shoulder and axilla were washed with a 7.5% povidone-iodine solution and rinsed with water. The surgical field was then prepared with 4% chlorhexidine gluconate and covered with sterile, non–iodine-impregnated drapes. All procedures were performed through a deltopectoral approach, with replacement of the scalpel blade after skin and subcutaneous incisions.

At the time of prosthesis implantation, three specimens were collected and placed in CultureSwab with Amies medium (Becton Dickinson Italia S.p.a., BD, Milan, Italy): two swabs from the prosthetic components (glenosphere “a” and humeral component “b”) and one periprosthetic tissue sample (“c”). These surfaces were selected because they represent the final implant interfaces exposed to the surgical field immediately before wound closure and may therefore reflect contamination potentially relevant to implant-associated infection, whereas periprosthetic tissue samples were collected from deep periarticular soft tissues adjacent to the implant site. An additional swab placed in the surgical field without tissue contact was collected during the same intraoperative stage as a negative control (“d”). Samples were processed for microbiological analysis within 1 h. Wound closure and dressing followed standard procedures. Patients were followed at 1, 2, and 4 weeks, and at 3, 6 and 12 months postoperatively to identify superficial or deep infections.

### 2.3. Sample Processing and Microbial Identification

Samples were analysed using both quantitative and qualitative approaches. After room-temperature sonication for 15 min (Sonorex Digitec DT 31/H, BANDELIN electronic GmbH & Co., Berlin, Germany) in 1 mL of sterile Amies medium, followed by brief vortexing, 100 μL of the sonication fluid was plated onto culture media for aerobic and anaerobic microorganisms. Colony counts were expressed as CFUs/mL by multiplying the number of recovered colonies by 10, since 100 μL of the initial 1 mL processed volume was plated. Specifically, Schaedler agar with 5% blood (BD, Milan, Italy), Nutrient Agar (NA; Merck KGaA, Darmstadt, Germany), Mannitol Salt Agar (MSA; Biokar Diagnostics, Allonne, France), and Sabouraud dextrose agar (Biokar Diagnostics) were used for anaerobes, aerobes, staphylococci, and fungi, respectively.

Plates were incubated at 37 °C for up to 21 days under anaerobic conditions using an anaerobic system (Gaspak EZ anaerobe pouch system kit, BD) and up to 10 days under aerobic conditions. Colony counts were expressed as colony-forming units (CFUs)/mL, and colonies were evaluated based on morphological characteristics (i.e., haemolysis, pigmentation, and shape). Selected colonies were Gram-stained, tested for catalase activity, and subcultured on NA as enriched medium or on selective/differential media (MSA and MacConkey agar). Final microbial identification was performed using matrix-assisted laser desorption/ionization time-of-flight mass spectrometry (MALDI-TOF; Bruker Daltonics GmbH, Bremen, Germany), with a score ≥ 2 deemed suitable.

### 2.4. Measures

Data collected included sex, age at surgery, BMI, and smoking status. Comorbidities were recorded as autoimmune disease, hypertension, cardiovascular disease, dyslipidemia, type 2 diabetes mellitus (DM), cancer, and dysthyroidism. Clinical variables included prior cortisone injections, surgical duration (skin incision to closure), indication for surgery (primary osteoarthritis, cuff tear arthropathy, massive rotator cuff tear, or fracture sequelae), antibiotic prophylaxis (cefazolin or vancomycin), and postoperative complications (infection, delayed wound healing, pyrexia, hemorrhage requiring transfusion, or hypersensitivity reactions).

Microbiological information was collected for both *C. acnes* and aerobic bacteria, including time to positivity (days of incubation), microbiological positivity status, bacterial load (CFUs/mL), and specimen location (a, b, c; see Section 2.2). Aerobic isolates were further classified by species (*S. epidermidis*, *S. capitis*, *S. hominis*, *S. lugdunensis*, *S. saprophyticus*, and *S. warneri*). *C. acnes* and aerobic bacteria were analysed separately due to their distinct microbiological characteristics and growth requirements. The comparison was performed to characterize the relative intraoperative burden of the predominant anaerobic and aerobic organisms recovered in shoulder arthroplasty, acknowledging their distinct biological and growth characteristics.

### 2.5. Statistical Analysis

Baseline demographic and clinical characteristics are reported at the patient level, whereas microbiological outcomes (bacterial load and positivity) were evaluated at the specimen level, accounting for within-patient clustering through mixed-effects models. Socio-demographic and clinical characteristics were analysed using proportions for categorical variables and means (SD) or medians (IQR) for continuous data. Differences according to *C. acnes* positivity and aerobe positivity were assessed independently using Fisher’s exact test or the Kruskal–Wallis test, as appropriate; patients could contribute to both microbiological groups. For microbiological analyses, specimens were treated as primary sampling units, and aerobic isolates were analysed separately by species in order to account for the independent detection of different aerobic microorganisms within the same specimen. Mixed-effects linear regression models with a patient-level random intercept and a non-parametric intra-cluster bootstrap were used to account for the hierarchical structure of the data and the non-normal distribution of bacterial load. Dependent variables were *C. acnes* or aerobe load, with covariates including sex, age, specimen location, BMI, hypertension, diabetes mellitus, cardiovascular disease, smoking, surgical duration, reason for surgery, and time to positivity; aerobe species were treated as fixed effects and added as additional covariates in aerobe load models. *C. acnes* and aerobe positivity were analysed using mixed-effects Cox proportional hazards models accounting for time to positivity and patient-level clustering, including the same covariates and previous positivity; aerobe species were additionally included in aerobe positivity models. Load models were assessed using the intraclass correlation coefficient (ICC) and positivity models using the penalized log-likelihood function. A *p*-value < 0.05 was considered statistically significant. All analyses were conducted using R statistical software (version 3.4.0; R Core Team).

## 3. Results

### 3.1. Patients’ Characteristics, Comorbidities, and Risk Factors

Table 1 presents the baseline and intervention characteristics of the sample, comparing patients by *C. acnes* positivity and aerobe positivity in intraoperative specimens. Overall, most of the sample included females (63.6%), and the mean age was 73.7 years (SD 7.54). The most represented comorbidities were hypertension (65.5%), cardiovascular disease (34.5%), and dyslipidemia (34.5%), followed by Diabetes Mellitus (DM, 16.4%), cancer (10.9%), autoimmune disease (7.3%), and dysthyroidism (7.3%). The mean BMI was 25.9 ± 4.43 kg/m^2^, and 10.9% of participants were current smokers. The most common reason for shoulder intervention was primary osteoarthritis (47.3%), followed by cuff tear arthropathy (CTA, 38.4%), massive rotator cuff tear (7.3%), and fracture sequelae (7.3%). The mean surgical length was 96 ± 21.2 min. *C. acnes* positivity (vs. *C. acnes* negativity) was associated with a statistically significantly greater proportion of males (56.7% vs. 12.0%; *p* < 0.001), while aerobe positivity (vs. aerobe negativity) was not associated with any of the understudy variables. During the follow-up period (1, 2, and 4 weeks; 3, 6 and 12 months), no cases of superficial or deep periprosthetic joint infection were observed. Three patients reported minor postoperative symptoms (e.g., ecchymosis or persistent shoulder pain), none of which fulfilled clinical or microbiological criteria for infection.

### 3.2. Microbiological Results: Bacterial Loads, Positivity Rates of Specimens, and Time of Positivity

**Figure 1:**
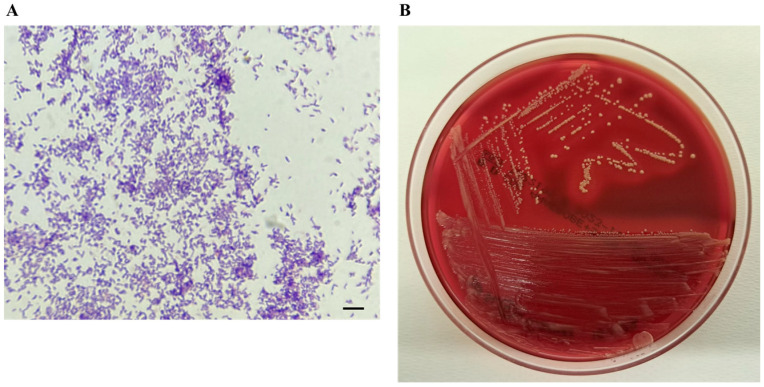
Small Gram-positive bacilli (**A**) isolated from a colony that grew on Schaedler agar, identified as *C. acnes* (magnification ×1000; scale bar = 5 mm), and its subculture with β-hemolysis (**B**).

**Figure 2:**
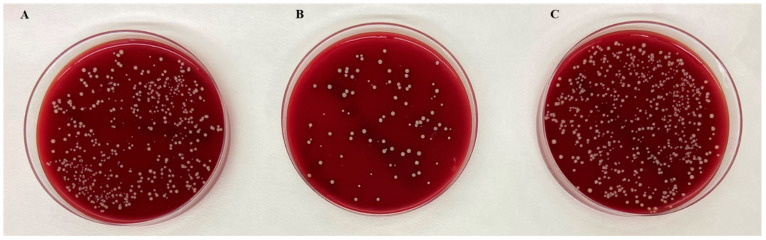
Representative images depicting the presence of anaerobic bacteria collected from specimens of the surgical field—swabs (**A**,**B**) and periprosthetic tissue (**C**)—after RTSA, and plated on Schaedler agar.

**Figure 3:**
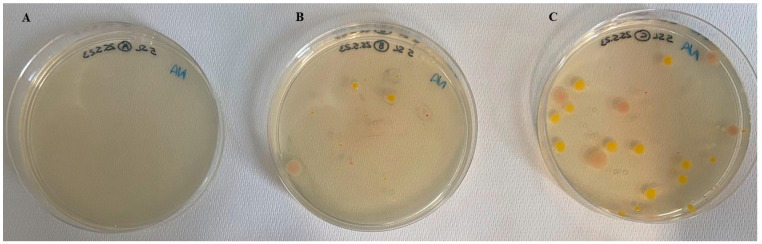
Representative images depicting the presence of aerobic bacteria collected from specimens of the surgical field—swabs (**A**,**B**) and periprosthetic tissue (**C**)—after RTSA, and plated on nutrient agar.

The preliminary macroscopic microbiological evaluation demonstrated that on Schaedler agar plates, small Gram-positive rods grew (Figure 1A), characterized, in some cases, by β-hemolysis (Figure 1B), whereas on NA plates, the most prevalent bacteria were Gram-positive and catalase-positive cocci. As presented in Figure 2 and Figure 3, the specimens—either swabs or periprosthetic tissue—collected at the end of RTSA showed the presence of either anaerobic or aerobic bacteria, seeded on Schaedler agar plates or NA, respectively. Under anaerobic incubation conditions, the only recovered and identified bacterium was *C. acnes*, whereas aerobic incubation allowed the growth of additional bacterial species. In the SAB plates used for fungal detection, as well as in all 55 negative controls, no growth was shown.

Table 2 reports the bacterial loads recovered from agar-plated specimens. *C. acnes* demonstrated mean loads corresponding approximately to 10^3^ CFU/mL, whereas aerobic bacteria showed substantially lower values, approximately 10^1^ CFU/mL, reaching a statistically significant difference. As stated, *C. acnes* was the only bacterium recovered under anaerobic incubation conditions, whereas CoNS and *E. coli* were recovered under aerobic incubation conditions. In particular, CoNS strains were distributed in species as follows: *S. epidermidis* (n = 11), *S. warneri* (n = 9), *S. hominis* (n = 8), *S. capitis* (n = 6), *S. saprophyticus* (n = 3), and *S. lugdunensis* (n = 2).

The next step was to evaluate the positivity rates across different specimen types for both anaerobic and aerobic bacteria (Table 3). A significant difference was observed in the overall sample set and in swab positivity, with anaerobic bacteria showing higher detection rates than aerobic ones (*p <* 0.05). Among specimens with *C. acnes* recovery, the mean time of achieving positivity was 5.99 days (95% CI: 5.33; 6.64), while among specimens with aerobe positivity, it was 2.91 days (95% CI: 2.32; 3.50).

When analyzed at the patient level (n = 55), 9 patients (16.4%) had no positive specimens, 32 (58.2%) had 1/3 positive samples, 11 (20.0%) had 2/3 positive samples, and 3 (5.5%) had 3/3 positive samples. Specifically for *C. acnes*, 30 patients (54.5%) had 1/3 positive samples, whereas none had 2/3 or 3/3 positive specimens. For aerobic bacteria, 22 patients (40.0%) had 1/3 positive samples and 10 (18.2%) had 2/3 positive samples; no patient had 3/3 positive aerobic specimens.

### 3.3. Factors Associated with Bacterial Loads

Results are presented as regression coefficients (b) with 95% confidence intervals. Table 4 shows factors associated with *C. acnes* load and aerobes load. A positive statistically significant association was found between *C. acnes* loads and male sex (b: 537.782, 95% CI: 85.387; 1750.397), age (b: 67.296, 95% CI: 28.047; 115.244), BMI (b: 107.595, 95% CI: 46.463; 164.437), dyslipidemia (b: 869.63, 95% CI: 95.688; 1869.499), smoke (b: 725.408, 95% CI: 27.775; 1218.473) and CTA as reason for surgery (b: 455.215, 95% CI: 22.82; 1126.326). A negative statistically significant association was observed between *C. acnes* loads and hypertension (b: −669.652, 95% CI: −1079.802; −261.178), DM (b: −1147.239, 95% CI: −2463.321; −270.71), cardiovascular disease (b: −801.563, 95% CI: −1588.253; −58.948), timing at positivity (b: −75.665, 95% CI: −113.309; −25.87), and massive rotator cuff tear as reason for surgery (b: −450.973, 95% CI: −875.274; −45.287).

Regarding aerobes, a positive statistically significant association was registered between aerobes load and length of surgery (b: 0.102, 95% CI: 0.015; 0.207), while a negative association was found with massive rotator cuff tear as reason for surgery (b: −2.7, 95% CI: −6.729; −0.134).

The random effects estimates revealed relevant differences between the two models (Table 4). In the model on *C. acnes* load, the between-patient variance (τ) and the within-patient variance (σ^2^) resulted in an intra-class correlation coefficient (ICC) of 0.41. This indicates that 41% of the total variance was attributable to differences between patients, suggesting substantial clustering and supporting the use of a multilevel modelling approach to account for the hierarchical structure of the data. An ICC of 0.41 for *C. acnes* load indicates that a substantial proportion of the variability in bacterial burden was attributable to differences between patients rather than differences between specimens within the same patient. This finding supports the relevance of patient-level characteristics in influencing *C. acnes* contamination. In contrast, the low ICC observed for aerobic bacteria (0.05) suggests that most variability occurred at the specimen level, with limited contribution from between-patient differences.

### 3.4. Factors Associated with Bacterial Positivity

Table 5 shows factors associated with *C. acnes* and aerobes positivity. A positive association was found between the hazard of *C. acnes* positivity and being male (HR: 19.01, 95% CI: 5.21; 69.32). Whereas, regarding aerobes, a negative association was registered between the hazard of aerobes positivity and the type of identified bacteria, i.e.*, S. lugdunensis* (HR: 0.12, 95% CI: 0.03; 0.51) and *S. saprophyticus* (HR: 0.18, 95% CI: 0.04; 0.42), and with previous positivity (HR: 0.13, 95% CI: 0.04; 0.42). This indicates that the identified factor was associated with a lower probability of intraoperative aerobic bacterial positivity.

For both outcomes, the inclusion of random effects significantly improved model fit, indicating meaningful clustering in the data. In both the mixed-effects Cox models, the penalized log-likelihood test was significant (*p* < 0.001), suggesting a better fit of the models that consider differences across patients. These results support the presence of between-patient variability in the hazard of each outcome.

## 4. Discussion

Contamination of the surgical field represents a major risk factor for PJI, a devastating complication. Although several studies have reported positive culture rates from skin samples after standard disinfection protocols or from joints following shoulder surgery, none have quantified the bacterial load of both anaerobic and aerobic organisms [3,4,5,7,9,15,20,21,24,25]. Therefore, the present study aimed to quantify microbial burden and culture positivity in specimens collected at the end of RTSA and to assess their association with patients’ comorbidities and lifestyle habits, to better characterize intraoperative surgical field contamination.

Matsen et al. (2020c) reported a reduction in both culture positivity and bacterial counts after skin disinfection [16]. In that study, the number of positive patients decreased for *C. acnes*, CoNS, and other bacteria; however, although *C. acnes* positivity declined, its bacterial load did not, and the composition of the “other bacteria” group was not specified [16]. Duvall et al. (2020) identified a bacterial load >10^3^ CFU/mL as a minimum threshold for *C. acnes* burden based on skin swab samples [12]. In the present study, bacterial loads were quantified in specimens collected at the end of RTSA [12]. On log_10_-transformed analysis, *C. acnes* demonstrated significantly higher loads than aerobic bacteria across specimen types, corresponding approximately to 10^3^ CFU/mL versus 10^1^ CFU/mL, respectively. These findings suggest distinct colonization patterns and reinforce the importance of sampling multiple surgical sites. While the higher intraoperative load of *C. acnes* may partly reflect its known cutaneous colonization density in the shoulder region, the absence of postoperative PJIs in our cohort prevents evaluation of a direct relationship between intraoperative bacterial burden and subsequent infection. These findings further support the potential importance of intraoperative strategies aimed at reducing bacterial burden, including optimized skin preparation and antiseptic irrigation protocols such as povidone-iodine application, which have been increasingly investigated in shoulder arthroplasty [7,8,26].

While bacterial load has been poorly investigated, several studies have reported microbial positivity rates in shoulder surgery. In our study, *C. acnes* showed a positivity rate of 42.4%, higher than that of aerobic bacteria (29.1%). These findings are consistent with previous reports, including a systematic review identifying a *C. acnes* positivity rate of 37.3% in biopsy samples following PJIs [27] and a study reporting *C. acnes* detection in 41.8% of patients undergoing primary shoulder arthroplasty [24].

Booker and colleagues (2017) reported that, in glenohumeral joint specimens, *C. acnes* was detected together with other bacilli and CoNS, predominantly *S. epidermidis*, *S. hominis*, and *S. warneri* [28]. Similar findings were observed in our study, with intraoperative specimens showing a higher prevalence of CoNS, mainly *S. epidermidis*, *S. warneri*, and *S. hominis*.

Male sex has been consistently identified as a risk factor for *C. acnes* positivity following shoulder arthroplasty, with higher rates of both culture positivity and infection compared with females [13,17,29,30,31]. This predisposition has been attributed to anatomical differences in the shoulder region, including a higher density of sebaceous glands and hair follicles in males. In our study, this association was confirmed and further linked to a higher *C. acnes* bacterial load in male patients (+537.872 CFUs). In contrast, no sex-related differences were observed for aerobic bacteria, either in positivity or bacterial count.

The role of preoperative cortisone injections in *C. acnes* contamination remains controversial, with increased risk reported when injections are performed close to surgery or after multiple administrations [32,33,34,35], while other studies found no association [36]. In our study, no association was observed between prior injections and *C. acnes* positivity or bacterial load, possibly due to the strict exclusion of patients who received injections within 6 months before surgery.

Previous studies have shown that time to culture positivity for *C. acnes* is influenced by bacterial load and can support interpretation of culture results. However, growth kinetics alone are insufficient to reliably distinguish contamination, colonization, and infection, and should therefore be interpreted with caution [18,37].

Shorter times to positivity have been associated with higher bacterial burden, whereas delayed growth has more often been linked to low-level contamination [22]. In our study, aerobic bacteria showed significantly shorter times to positivity (2.91 days) compared with *C. acnes* (5.99 days), likely reflecting intrinsic differences in growth kinetics, as aerobic and facultative anaerobic organisms replicate more rapidly under routine incubation conditions. In contrast, *C. acnes* is a slow-growing anaerobic bacterium; however, in our cohort, higher *C. acnes* loads (~10^3^ CFU/mL) were associated with shorter times to detection. This indicates that, although growth rates differ between species, increased bacterial burden may accelerate time to positivity even in slow-growing organisms. Therefore, time to positivity should be interpreted considering both intrinsic microbial characteristics and quantitative culture results.

We observed significantly higher *C. acnes* bacterial loads in patients undergoing RTSA for primary osteoarthritis or CTA compared with those treated for massive rotator cuff tear or fracture sequelae, with CTA showing higher loads than primary osteoarthritis. Although samples were collected at the end of the surgical procedure, these findings are consistent with previous reports suggesting a possible involvement of *C. acnes* in shoulder osteoarthritis [5,24]. Nevertheless, this association should be interpreted cautiously and regarded as descriptive, as differences in bacterial load—particularly between massive rotator cuff tear and CTA—may reflect variations in the local biological environment rather than a direct pathogenic role of *C. acnes*.

Patient-related and lifestyle factors may influence both bacterial positivity and the risk of developing PJIs [3,6,7,8,15,19,25]. Age has been proposed as a potential risk factor, with higher positivity rates reported in patients over 75 years; in our study, *C. acnes* load increased by approximately 67.3 CFUs for each additional year of patient age. However, conflicting results have also been described in the literature [9,29,33,38]. Similarly, BMI—previously associated with postoperative complications—was positively associated with increased *C. acnes* load in our cohort [29,38].

A recent review by Saad et al. highlighted patient-related risk factors for PJIs, including surgery duration, comorbidities (e.g., DM, Parkinson’s disease, HIV, and HCV infections), and lifestyle habits such as smoking [8,29]. Consistent with these findings, we observed an association between *C. acnes* load and smoking. In contrast, hypertension, DM, and cardiovascular disease appeared to be inversely associated with *C. acnes* load, possibly reflecting the limited number of affected patients in our cohort. Therefore, these findings should be interpreted cautiously and considered preliminary, as residual confounding and subgroup imbalance related to the limited sample size may have influenced the observed associations. Compared with *C. acnes*, surgical duration showed a stronger association with the presence of aerobic bacteria and with the detection of less common CoNS species, such as *S. lugdunensis* and *S. saprophyticus*. Given the limited available literature, these observations warrant confirmation in larger patient cohorts.

Recent evidence indicates that the diagnosis of shoulder PJI results from a complex interplay of patient- and surgeon-related factors and cannot be inferred from microbiological findings alone [39]. This study has several limitations, including the limited sample size, consistent with the exploratory nature of the study and the inability to draw conclusions on risk factors for PJI, as the analysis focused on intraoperative surgical field contamination—defined by microbial presence, burden, and time to culture positivity—at the end of the procedure, and *C. acnes* phenotypes were not distinguished. In addition, no postoperative PJIs were observed during follow-up, which precluded assessment of a direct relationship between intraoperative bacterial burden and subsequent infection. Analyses involving aerobic bacterial species should be interpreted cautiously, since multiple aerobic microorganisms could be identified within the same specimen. Nevertheless, a major strength of this study is the assessment of not only culture positivity but also bacterial load in the surgical field during shoulder surgery, which may help clarify the significance of unexpected positive cultures, often regarded as false positives [40].

## 5. Conclusions

Intraoperative bacterial contamination of the surgical field during RTSA is frequent and characterized by differences in bacterial load and time to positivity between *C. acnes* and aerobic bacteria. Male sex, age, BMI, smoking, and surgical indication were associated with higher *C. acnes* burden, whereas surgical duration was associated with aerobic bacterial load. Quantitative assessment of bacterial burden, beyond culture positivity, may improve the interpretation of unexpected positive intraoperative cultures in shoulder arthroplasty.

## Figures and Tables

**Table 1:** Descriptive of baseline and intervention characteristics of the sample, overall and by *C. acnes* positivity and aerobe positivity.

	Overall	*C. acnes* Negative	*C. acnes* Positive	*p*-Value	Aerobe Negative	Aerobe Positive	*p*-Value
	(N = 55)	(N = 25)	(N = 30)		(N = 29)	(N = 26)	
**Sex**				<0.001			0.575
Female	35 (63.6%)	22 (88.0%)	13 (43.3%)		17 (58.6%)	18 (69.2%)	
Male	20 (36.4%)	3 (12.0%)	17 (56.7%)		12 (41.4%)	8 (30.8%)	
**Age**				0.421			0.120
Mean (SD)	73.7 (7.54)	75.0 (6.17)	72.7 (8.48)		75.0 (7.44)	72.3 (7.55)	
Median [Q1. Q3]	76.0 [68.5. 79.0]	76.0 [73.0. 79.0]	75.0 [67.3. 78.0]		77.0 [71.0. 79.0]	74.0 [64.3. 77.0]	
Min–Max	52.0–85.0	63.0–83.0	52.0–85.0		52.0–85.0	57.0–83.0	
**BMI**				0.161			0.953
Mean (SD)	25.9 (4.43)	26.6 (3.99)	25.6 (4.76)		26.1 (5.26)	25.7 (3.36)	
Median [Q1. Q3]	25.6 [23.4. 27.6]	26.6 [23.4. 29.1]	24.2 [23.2–26.8]		25.6 [22.5. 28.4]	25.0 [23.7. 27.4]	
Min–Max	15.9–39.8	20.4–35.2	15.9–39.8		15.9–39.8	20.2–35.2	
**Hypertension**				0.404			1
No	19 (34.5%)	7 (28.0%)	12 (40.0%)		10 (34.5%)	9 (34.6%)	
Yes	36 (65.5%)	18 (72.0%)	18 (60.0%)		19 (65.5%)	17 (65.4%)	
**Cardiovascular disease**				0.163			0.273
No	36 (65.5%)	19 (76.0%)	17 (56.7%)		21 (72.4%)	15 (57.7%)	
Yes	19 (34.5%)	6 (24.0%)	13 (43.3%)		8 (27.6%)	11 (42.3%)	
**Dyslipidemia**				1			1
No	36 (65.5%)	16 (64.0%)	20 (66.7%)		19 (65.5%)	17 (65.4%)	
Yes	19 (34.5%)	9 (36.0%)	10 (33.3%)		10 (34.5%)	9 (34.6%)	
**DM**				0.064			0.475
No	46 (83.6%)	18 (72.0%)	28 (93.3%)		23 (79.3%)	23 (88.5%)	
Yes	9 (16.4%)	7 (28.0%)	2 (6.7%)		6 (20.7%)	3 (11.5%)	
**Cancer**				1			0.672
No	49 (89.1%)	22 (88.0%)	27 (90.0%)		25 (86.2%)	24 (92.3%)	
Yes	6 (10.9%)	3 (12.0%)	3 (10.0%)		4 (13.8%)	2 (7.7%)	
**Dysthyroidism**				0.617			1
No	51 (92.7%)	24 (96.0%)	27 (90.0%)		27 (93.1%)	24 (92.3%)	
Yes	4 (7.3%)	1 (4.0%)	3 (10.0%)		2 (6.9%)	2 (7.7%)	
**Autoimmune disease**				0.320			1
No	51 (92.7%)	22 (88.0%)	29 (96.7%)		27 (93.1%)	24 (92.3%)	
Yes	4 (7.3%)	3 (12.0%)	1 (3.3%)		2 (6.9%)	2 (7.7%)	
**Smoke**				1			1
No	49 (89.1%)	22 (88.0%)	27 (90.0%)		26 (89.7%)	23 (88.5%)	
Yes	6 (10.9%)	3 (12.0%)	3 (10.0%)		3 (10.3%)	3 (11.5%)	
**Injections**				0.583			0.785
No	23 (41.8%)	9 (36.0%)	14 (46.7%)		13 (44.8%)	10 (38.5%)	
Yes	32 (58.2%)	16 (64.0%)	16 (53.3%)		16 (55.2%)	16 (61.5%)	
**Surgical length**							
Mean (SD)	96.0 (21.1)	95.2 (17.5)	96.6 (23.9)	0.852	97.5 (22.6)	94.3 (19.6)	0.691
Median [Q1. Q3]	90.0 [82.0. 109]	92.0 [82.0. 110]	90.0 [82.5. 107]		90.0 [84.0. 111]	91.0 [80.5. 105]	
Min–Max	55.0–160	60.0–123	55.0–160		55.0–160	60.0–139	
**Reason for surgery**				0.674			0.935
Primary osteoarthritis	26 (47.3%)	14 (56.0%)	12 (40.0%)		15 (51.7%)	11 (42.3%)	
Cuff tear arthropathy	21 (38.2%)	8 (32.0%)	13 (43.3%)		10 (34.5%)	11 (42.3%)	
Massive rotator cuff tear	4 (7.3%)	1 (4.0%)	3 (10.0%)		2 (6.9%)	2 (7.7%)	
Fracture sequelae	4 (7.3%)	2 (8.0%)	2 (6.7%)		2 (6.9%)	2 (7.7%)	
**Antibiotic prophylaxis**				0.242			1
Cefazolin	52 (94.5%)	25 (100%)	27 (90.0%)		27 (93.1%)	25 (96.2%)	
Vancomycin	3 (5.5%)	0 (0%)	3 (10.0%)		2 (6.9%)	1 (3.8%)	
**Post-Surgery complications**				0.590			0.603
No	49 (89.1%)	22 (88.0%)	27 (90.0%)		26 (89.7%)	23 (88.7%)	
Yes	3 (5.5%)	2 (8.0%)	1 (3.3%)		1 (3.4%)	2 (7.7%)	
Missing	3 (5.5%)	1 (4.0%)	2 (6.7%)		2 (6.9%)	1 (3.8%)	

Abbreviations: Body Mass Index, BMI; Diabetes Mellitus, DM.

**Table 2:** Bacterial counts, as CFUs/mL, recorded on the three collected samples from the surgical area immediately after RTSA, for anaerobic—*C. acnes*—and aerobic bacteria, expressed as mean ± standard errors of the means (SEM).

	Bacterial Loads as CFUs/mL		Statistical Analysis	
Specimens	*C. acnes*	Aerobic Bacteria	Paired *t*-Test	C.I. 95%
Swabs (a,b)	1476 ± 469.8	61.17 ± 15.38	*p* = 0.0074	569.8 to 3508
Periprosthetic tissues (c)	1028 ± 373.3	78.89 ± 27.39	*p* = 0.0378	56.38 to 1842
Total of samples	1380 ± 377.6	65.37 ± 13.35	*p* = 0.0056	389.8 to 2240

**Table 3:** Bacterial detection rates recorded on the three collected samples from the surgical area immediately after RTSA, for anaerobic—*C. acnes*—and aerobic bacteria, expressed as positive specimens and percentages (%).

	Bacterial Positivity (%)		Statistical Analysis
Specimens; Number	*C. acnes*	Aerobic Bacteria	Fisher Exact Test
Swabs (a,b); n = 110	45 (40.9%)	30 (27.3%)	*p* = 0.0157
Periprosthetic tissues (c); n = 55	25 (45.5%)	18 (32.7%)	*p* = 0.2409
Total of samples; n = 165	70 (42.4%)	48 (29.1%)	*p* = 0.0461

**Table 4:** Mixed-effects linear regression models for factors associated with *C. acnes* and aerobic bacterial load. Reported values represent regression coefficients (b) with corresponding 95% confidence intervals (2.5–97.5%). Random effects parameters include between-patient variance (τ), within-patient variance (σ^2^), and intraclass correlation coefficient (ICC).

	*C. acnes* Load			Aerobes Load		
Variable	b	2.5%	97.5%	b	2.5%	97.5%
**Sex**						
Female	ref.			ref.		
Male	537.872	85.387	1750.397	−2.575	−6.09	0.026
**Age (per year)**	67.296	28.047	115.244	0.034	−0.337	0.323
**Location**						
a/b	ref.			ref.		
c	186.75	−251.061	747.768	−1.183	−4.478	2.259
**BMI (per kg/m^2^)**	107.595	46.463	164.437	−0.195	−0.786	0.27
**Hypertension**	−669.652	−1079.802	−261.178	−1.033	−4.823	2.543
**DM**	−1147.239	−2463.321	−270.71	−1.286	−3.932	0.739
**Dyslipidemia**	869.63	95.688	1869.499	1.034	−3.069	6.464
**Cardiovascular disease**	−801.563	−1588.253	−58.948	3.177	−0.587	7.65
**Smoke**	725.408	27.775	1218.473	−4.042	−8.38	−0.927
**Intervention duration (per minute)**	0.127	−5.696	5.602	0.102	0.015	0.207
**Reason for intervention**						
Primary osteoarthritis	ref.			ref.		
Cuff tear arthropathy	455.215	22.82	1126.326	0.046	−3.373	4.119
Massive rotator cuff tear	−450.973	−875.274	−45.287	−2.7	−6.729	−0.134
Fracture sequelae	−265.567	−586.743	37.356	7.044	−2.424	18.83
**Microorganism**						
*S. epidermidis*				ref.		
*S. capitis*				−8.167	−12.689	−4.752
*S. hominis*				2.483	−3.47	8.804
*S. lugdunensis*				0.581	−4.398	5.436
*S. saprophyticus*				0.521	−3.889	4.801
*S. warneri*				4.667	−2.688	13.251
**Timing of positivity (per day)**	−75.665	−113.309	−25.87	1.564	−3.838	6.815
**Random effects**						
Between-patient variance (τ)	2,604,611.066	283,700.237	13,668,330	38.283	0.558	141.642
Within patient variance (σ^2^)	3,329,705.755	233,353.392	5,255,392	648.684	230.747	1153.533
Intraclass Correlation Coefficient (ICC)	0.404	0.111	0.934	0.051	0.002	0.127

Abbreviations: Body Mass Index, BMI; Diabetes Mellitus, DM. Note: bolded if *p* < 0.05.

**Table 5:** Factors associated with *C. acnes* and aerobe positivity.

	*C. acnes* Positivity			Aerobe Positivity		
	HR	95% CI	*p*	HR	95% CI	*p*
**Sex**						
Female	ref.			ref.		
Male	19.01	5.21–69.32	<0.001	0.63	0.30–1.34	0.232
**Age (year)**	1.01	0.93–1.09	0.869	0.98	0.94–1.03	0.496
**Location**						
a/b	0.66	0.39–1.11	0.119	0.86	0.47–1.57	0.622
c	ref.			ref.		
**BMI (per kg(m^2^)**	1.04	0.86–1.25	0.677	1.00	0.91–1.11	0.933
**Hypertension**	0.22	0.06–0.82	0.025	1.12	0.49–2.59	0.784
**DM**	0.13	0.01–1.16	0.068	0.53	0.16–1.75	0.297
**Dyslipidemia**	0.85	0.20–3.56	0.820	1.32	0.63–2.75	0.466
**Cardiovascular disease**	1.69	0.49–5.82	0.409	1.83	0.90–3.72	0.097
**Smoke**	2.20	0.27–18.04	0.464	1.04	0.36–3.02	0.949
**Intervention duration (per minute)**	2.90	0.79–10.62	0.107	1.33	0.63–2.77	0.453
**Reason for intervention**						
Primary osteoarthritis	ref.			ref.		
Cuff tear arthropathy	1.98	0.14–28.93	0.617	1.35	0.37–4.94	0.647
Massive rotator cuff tear	2.44	0.22–27.61	0.471	1.82	0.49–6.79	0.371
Fracture sequelae	0.99	0.96–1.02	0.573	1.00	0.98–1.02	0.756
**Microorganism**						
*S. epidermidis*				ref.		
*S. capitis*				0.42	0.17–1.02	0.057
*S. hominis*				0.47	0.20–1.11	0.085
*S. lugdunensis*				0.12	0.03–0.51	0.004
*S. saprophyticus*				0.18	0.05–0.60	0.006
*S. warneri*				0.61	0.28–1.35	0.221
**Previous positivity**	0.67	0.33–1.36	0.264	0.13	0.04–0.42	0.001
**N**	55			55		
**Observations**	196			1415		
**Random effects**						
**Penalized log-likelihood**						
**χ^2^ (df)**	218.2 (34.9)			59.6 (24.4)		
***p***	<0.001			<0.001		
**Akaike Information Criterion (AIC)**	148.5			10.85		

Abbreviations: Body Mass Index, BMI; Diabetes Mellitus, DM. Note: bolded if *p* < 0.05.

## Data Availability

All data generated or analysed during this study are included in this article.
